# The Promise of Magnetic Resonance Imaging in Radiation Oncology Practice in the Management of Brain, Prostate, and GI Malignancies

**DOI:** 10.1200/GO.21.00366

**Published:** 2022-05-24

**Authors:** Shashank Srinivasan, Archya Dasgupta, Abhishek Chatterjee, Akshay Baheti, Reena Engineer, Tejpal Gupta, Vedang Murthy

**Affiliations:** ^1^ Department of Radiation Oncology, Tata Memorial Centre, Homi Bhabha National Institute, Mumbai, India; ^2^ Department of Radiodiagnosis, Tata Memorial Centre, Homi Bhabha National Institute, Mumbai, India

## Abstract

Magnetic resonance imaging (MRI) has a key role to play at multiple steps of the radiotherapy (RT) treatment planning and delivery process. Development of high-precision RT techniques such as intensity-modulated RT, stereotactic ablative RT, and particle beam therapy has enabled oncologists to escalate RT dose to the target while restricting doses to organs at risk (OAR). MRI plays a critical role in target volume delineation in various disease sites, thus ensuring that these high-precision techniques can be safely implemented. Accurate identification of gross disease has also enabled selective dose escalation as a means to widen the therapeutic index. Morphological and functional MRI sequences have also facilitated an understanding of temporal changes in target volumes and OAR during a course of RT, allowing for midtreatment volumetric and biological adaptation. The latest advancement in linear accelerator technology has led to the incorporation of an MRI scanner in the treatment unit. MRI-guided RT provides the opportunity for MRI-only workflow along with online adaptation for either target or OAR or both. MRI plays a key role in post-treatment response evaluation and is an important tool for guiding decision making. In this review, we briefly discuss the RT-related applications of MRI in the management of brain, prostate, and GI malignancies.

## INTRODUCTION

Magnetic resonance imaging (MRI) is an imaging modality on the basis of the principle of nuclear magnetic resonance. As hydrogen atoms constitute the major share of the human body, it enables the use of nuclear magnetic resonance for clinical imaging.^1^ Since the first human MRI images were acquired in 1977, MRI has evolved rapidly with the development of relatively faster image acquisition, increase in magnetic field strength, and improved image processing techniques.^2,3^ The ability of noninvasive characterization of internal anatomy attributed to better soft-tissue clarity and the development of functional sequences to capture the internal physiology have facilitated numerous clinical applications of MRI. In clinical practice, MRI plays a pivotal role in diagnosis, disease staging, treatment planning, response monitoring, and surveillance after treatment completion.

**CONTEXT**

**Key Objective**
Magnetic resonance imaging (MRI) constitutes an integral role in contemporary oncology practice. This review was aimed at discussing radiotherapy (RT)–related applications of MRI in the context of brain, prostate, and GI malignancies.
**Knowledge Generated**
MRI plays a crucial role in decision making, RT planning and delivery, and response evaluation. MRI protocols including standardized sequences and slice thickness are required for target volume and organs at risk delineation for individual disease sites. The advent of MRI-linear accelerator (MR-Linac) provides opportunities for precise treatment delivery and real-time treatment adaptation, potentially improving therapeutic ratio. The merits of using MR-Linac are being investigated in ongoing clinical trials, with recent evidence suggesting reduction in acute toxicities with prostate stereotactic body radiotherapy delivered on MR-Linac.
**Relevance**
This review summarizes the current and expanding role of MRI in brain, prostate, and GI malignancies to allow optimum integration with RT practice.

**FIG 1:**
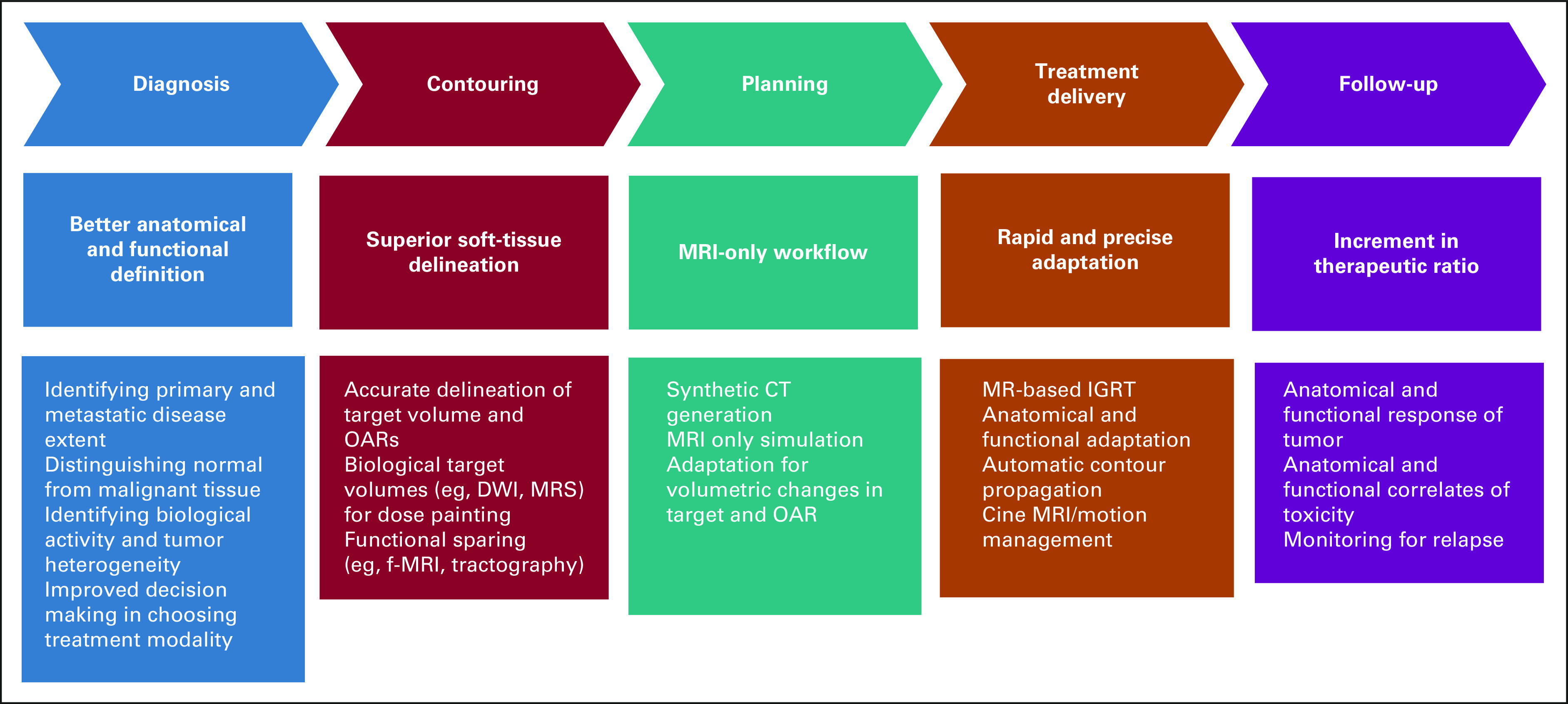
ROLE of MRI in radiation oncology workflow. CT, computed tomography; DWI, diffusion-weighted imaging; f-MRI, functional MRI; IGRT, image-guided radiotherapy; MRI, magnetic resonance imaging; MRS, spectroscopy; OAR, organs at risk.

Radiation oncology practice is deeply intertwined with imaging, primarily aiding in target volume and organs at risk (OAR) delineation and computing radiotherapy (RT) doses in the planning process (Fig 1). With the advancement and development of conformal and high-precision techniques such as intensity-modulated radiotherapy, stereotactic radiosurgery, stereotactic body radiotherapy (SBRT), and particle beam therapy, the need for imaging modalities with better anatomical information has become essential.^4,5^ Insights from molecular imaging such as positron emission tomography (PET) and functional MRI have paved the way toward dose painting.^6^ Similarly, midtreatment volumetric and biological adaptation using morphological and functional MRI sequences accounting for changes to the target volumes and OAR can help improve the therapeutic ratio in the form of adaptive RT.^7^ Image-guided radiotherapy involving online imaging before treatment delivery has improved the precision and accuracy of treatment delivery.^8^ The traditional platforms for image-guided radiotherapy involve computed tomography (CT) built in the treatment unit. The integration of a compatible MRI scanner with a linear accelerator (linac) device has successfully led to an MR-linear accelerator (MR-Linac), which had been introduced in clinical practice, popularly known as MRI-guided radiotherapy (MRgRT).^9,10^ In this review, we briefly discuss the clinical applications of MRI in the management of brain, prostate, and GI malignancies from the perspective of radiation oncologists.

## ROLE OF MRI IN CONTEMPORARY RADIATION ONCOLOGY PRACTICE

### CNS Malignancies

#### Rationale and parameters.

MRI forms an indispensable part of the contemporary management of CNS tumors. The superior soft-tissue resolution, true multiplanar imaging capability, and the capacity for innate multiparametric functional imaging such as diffusion-weighted imaging (DWI), intravoxel incoherent motion, perfusion imaging, chemical exchange saturation transfer, and blood oxygenation level-dependent predicate a decisive advantage for MRI over all other forms of cross-sectional imaging in this regard. Typically, field strengths of 1.5-3T are used for routine imaging of CNS tumors, with no clear advantage of the higher field strength in clinical practice.^11^ Imaging for diagnosis and response assessment usually requires axial T1 (pregadolinium and postgadolinium), axial T2, and fluid-attenuated inversion recovery (FLAIR) sequences at minimum, with DWI, perfusion-weighted imaging, and MR spectroscopy assisting initial diagnosis and later distinction between tumor progression and pseudoprogression.^12,13^ Thin slice (1-2 mm width) volumetric sequences such as 3-dimensional fast spoilt gradient (3D FSPGR) and 3D FLAIR offer the advantages of rapid acquisition, good image reconstruction, and increased lesion detection rates, facilitating accurate delineation and ultra high-precision RT planning.^14-16^ Additional sequences such as steady-state free precession sequences (CISS/FIESTA sequence as used by vendors) and fat suppression sequences are often used in the delineation of skull base tumors or targets in close relation to the brain stem and cranial nerves (eg, chordoma, chondrosarcoma, meningioma, and schwannoma) because of anatomical clarity. Acquisition of thin-slice MRI sequences aids in accurate estimation of lesion size as higher slice width can lead to overestimation of the delineated structures because of interslice interpolation.^17^ Initial imaging should ideally be performed no later than 72 hours after surgery, else delayed by 2 weeks to avoid obfuscation of imaging findings by blood products and postoperative changes.^12^ Imaging follow-up is usually performed at 4-6 weeks after RT conclusion, at completion of planned adjuvant therapy, and on clinical suspicion of progression or symptomatic worsening.^18^ Diffusion tensor imaging has proven beneficial in detecting radiation-induced demyelination and axonal degeneration,^19,20^ resulting in neurocognitive deterioration providing a window of changes before clinical manifestation and early interventions.

#### Clinical applications.

**FIG 2:**
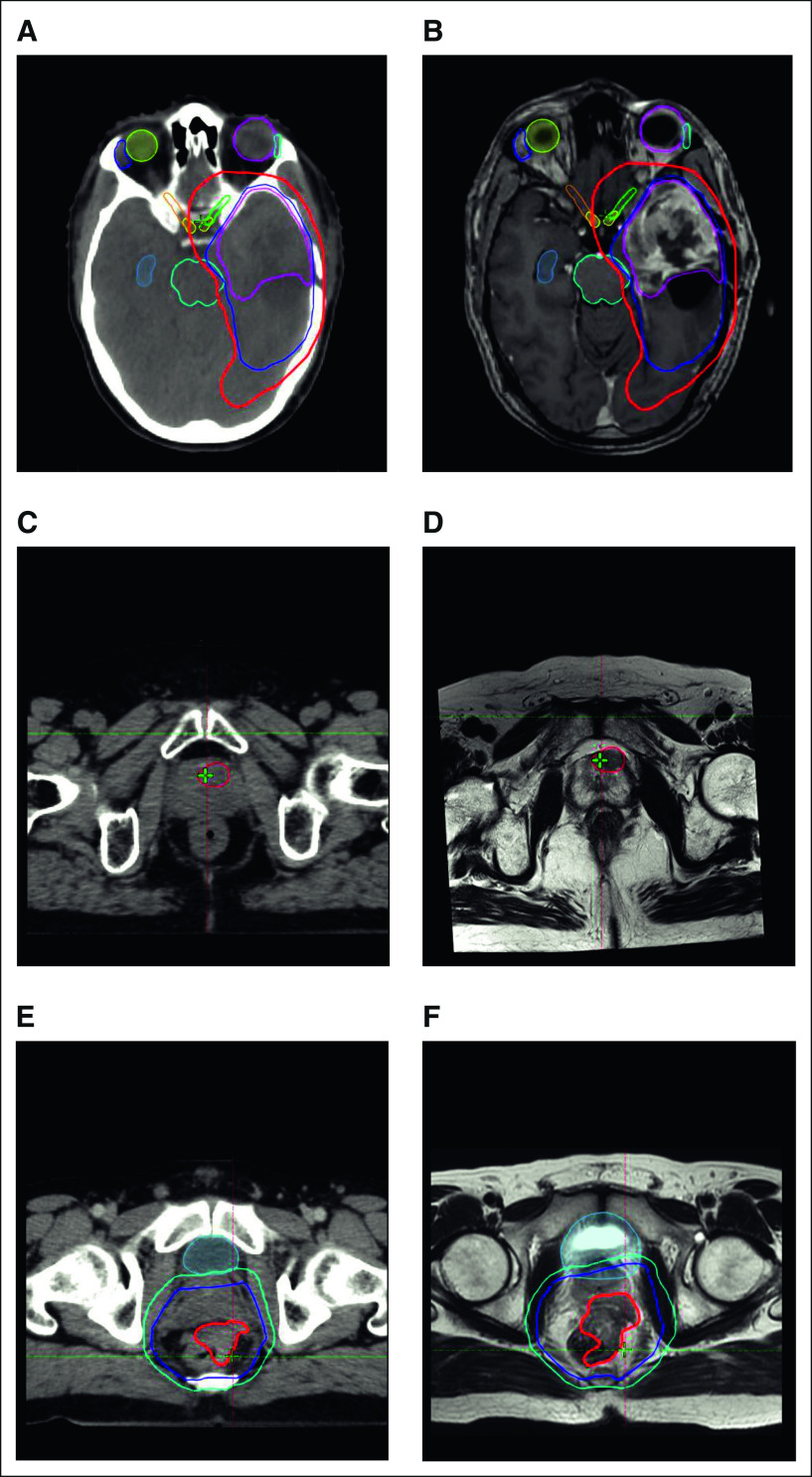
Composite diagram of CT and MRI used for radiation planning for brain, prostate, and GI malignancies. Representative CT and MRI scans acquired during radiation planning process. (A) Axial CT and (B) corresponding axial MRI T1-weighted contrast-enhanced sequence for a patient with glioblastoma. The target volumes are seen over the left temporal lobe: GTV (magenta), CTV (blue), and PTV (red). The extension of PTV in the basifrontal region was a result of expansion from extension of CTV in the superior slices (not seen in this image). (C) Axial CT and (D) corresponding axial T2W MRI for a patient with prostate cancer. Dominant intraprostatic lesion clearly visualized (in red) on the MRI, whereas it could not be discerned on the planning CT images, thus facilitating dose escalation to the DIL. (E) Axial CT and (F) corresponding axial T2W MRI for a patient with locally advanced rectal adenocarcinoma. Target volumes include GTV (red), CTV (purple), and PTV (light blue). The extension of the gross disease into the prostate could be accurately seen only on the MRI. CT, computed tomography; CTV, clinical target volume; DIL, dominant intraprostatic lesion; MRI, magnetic resonance imaging; PTV, planning target volume.

MRI forms a crucial part of target delineation in primary and metastatic tumors in the CNS. Complementary sequences (T1 postcontrast MRI to delineate the enhancing residual and areas of leptomeningeal dissemination and T2/FLAIR to distinguish areas of infiltrative and nonenhancing tumors) are typically used for comprehensive delineation.^21,22^ MRI image fusion is typically achieved with a high degree of accuracy in the brain and is facilitated by the presence of rigid bony anatomical markers and limited movement of the brain within the calvarium. Rigid registration algorithms are usually sufficient for image fusion.^23,24^ The accuracy of image fusion is confirmed by matching standard anatomical references (clinoid processes, bony sella, tentorium cerebelli, and vertebral artery). The standard imaging protocols share the common caveat of inability to distinguish between infiltrative disease and vasogenic edema with reliability while delineating the clinical target volume. Additionally, contemporary recommendations on MRI anatomy facilitate delimitation of the clinical target volume with respect to anatomical barriers and provide useful adjuncts as practice shifts to MRI-based planning and delivery in the current decade^25^ (Figs 2A and 2B).

The target volumes (postoperative cavity, surrounding infiltrative disease/vasogenic edema) in gliomas can undergo significant changes during the course of RT.^26^ The dynamic changes offer the opportunity to modify elective target volumes as areas of putative tumor tissue reduce in responders to therapy. Similarly, special consideration is to be given to cystic tumors such as craniopharyngioma, wherein a significant proportion of patients have been reported to have changes in cyst dimensions requiring treatment modifications during a course of fractionated RT spanning over several weeks.^27^ Automatic contour propagation facilitates real-time adaptation with accurate delineation in this regard.^28^ The significant challenges in this regard remain the accurate determination of the shifting tumor-normal tissue interface on anatomic and functional imaging. Limited evidence does suggest that it may be possible to distinguish the former from the latter through higher-order radiomic analysis, allowing one to potentially de-escalate RT in areas of response (reducing radiation necrosis and corticosteroid and bevacizumab usage rates) while intensifying therapy in voxels suggestive of radioresistance.^29^ Although anatomical variations in the target and OARs are demonstrated during the course of fractionated RT, the clinical merits either in terms of improving disease control or reducing toxicities need to be proven from prospective clinical trials.

Functional MRI constitutes a profusion of sequences that allow for comprehensive biological assessment of a tumor and is emerging as a useful adjunct for optimizing treatment. It has long been known that metabolic abnormalities (increased choline, reduced n-acetyl aspartate, and increased lipid lactate) exist beyond the tumor.^30^ In addition, areas with increased choline: n-acetyl aspartate ratios have also been found to correlate with adverse outcomes and are currently being targeted for dose escalation in clinical trials.^31^ Additional emerging areas for assessing tumor response include chemical exchange saturation transfer MRI, which can potentially detect both early response and tumor progression without the necessity for exogenous contrast.^32^ Noncontrast-based studies providing a combination of both diffusion and perfusion matrices such as intravoxel incoherent motion^33^ provide another powerful tool for assessing response to treatment.^34^ It is quite likely that such protocols in isolation or combination will provide opportunities for real-time biological adaptation in the setting of proliferation of MRI-based RT delivery systems with rapid onboard functional imaging capability. Table 1 shows selected studies for the role of MRI related to RT for brain tumors.

**TABLE 1:**
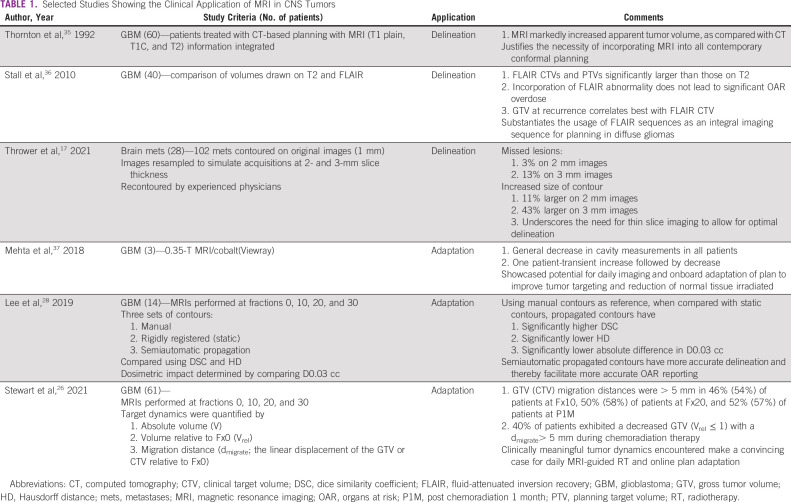
Selected Studies Showing the Clinical Application of MRI in CNS Tumors

### Prostate Cancer

#### Rationale and parameters.

MRI is central to external beam radiotherapy planning for prostate cancer. Multiparametric MRI (mp-MRI) is the recommended technique in prostate cancer combining anatomical with functional imaging. This includes a high-resolution T2-weighted imaging (T2WI) with at least two functional MRI techniques. DWI, dynamic contrast-enhanced (DCE) perfusion imaging, and occasionally MR spectroscopy are commonly used. 3-T scanners are preferred to 1.5-T as they provide a higher signal to noise ratio allowing for better structural and functional details.^38^ The study is performed on an external-phased array coil; using an endorectal coil does not offer significant benefit and may be avoided. Thin-slice (3 mm without interslice gap) T2W images with a small field of view (FOV) are used to depict prostate anatomy.^39^ The high spatial resolution enables accurate assessment of extracapsular extension and seminal vesicle invasion. Addition of DWI aids in differentiating malignant from benign lesions with the former having restricted diffusion.^40^

#### Clinical applications.

MRI plays a pivotal role in target volume delineation in prostate RT. The apex and base of the prostate are often poorly visualized on CT. MRI helps to differentiate the prostatic apex from the genitourinary diaphragm and the penile bulb and the base of the prostate from the bladder wall.

One of the biggest challenges for the use of MRI for volume delineation is the accuracy of CT and MRI coregistration as pelvic organs have nonrigid anatomy. The CT and T2W MRI images are fused using rigid automatic registration algorithm (on the basis of bony landmarks) and thereafter can be adjusted manually. For patients who have gold fiducial seeds implanted before the simulation scans, the images are aligned on the basis of the midpoint of the gold seeds. The prostate is then evaluated in all three planes to ensure precise anatomical superimposition. The presence of gold fiducial markers offers advantages for image coregistration as it overcomes the issue of accurately defining bony landmarks on MRI. Wegener et al^41^ showed that with the use of gold markers, the CT and MRI matching precision was within 2 mm.

Some of the issues precluding accurate CT-MRI coregistration are variations in rectal and bladder filling and patient position for the two scans with CT scan usually performed on a flat couch with knee support and the MRI being performed on a rounded table top unless using a dedicated MRI simulator. Chen et al^42^ showed that significant fusion uncertainties of > 4 mm were seen in 8.6% (anteroposterior direction) and 11.4% (superoinferior direction) of the patients with higher difference when scans were performed on different days. Patients with gold seed markers in situ had less prostate fusion uncertainties.

Prostate volume, as delineated on MRI, is nearly 30%-40% smaller than that delineated on CT with less interobserver variability.^43-47^ The maximum discrepancy in the two volumes is in the region of the prostatic apex and at the base of the seminal vesicles. Reduction in the target volumes potentially translates into better OAR sparing. Steenbakkers et al^48^ showed that the rectal wall for CT-delineated prostate plans received 5.1 Gy higher equivalent uniform dose, and the penile bulb received 11.6 Gy higher mean dose than the MRI-delineated prostate plans. This also meant that allowing for the same rectal wall dose, the planning target volume (PTV) dose could be escalated from 78 Gy to 85 Gy using plans on the basis of MRI delineation of the prostate. Ali et al^49^ compared intensity-modulated radiotherapy plans generated using CT-MRI delineation versus CT alone and found a statistically significant reduction in dose to the bladder and rectum with an approximately 22% reduction in Gr2 GU toxicity for CT-MRI patients, as compared with CT alone.

Patients with prostate cancer who develop local recurrence tend to do so at the site of the dominant intraprostatic lesion (DIL).^50^ mp-MRI provides excellent visualization of the DIL and has allowed the escalation of dose to the DIL to > 90 Gy^51,52^ (Figs 2C and 2D). The FLAME trial randomly assigned patients with localized prostate cancer to either standard RT (77 Gy to the entire prostate) or an additional integrated focal boost to the DIL to a dose of upto 95 Gy. The dose-escalation arm showed a superior biochemical disease-free survival (92% *v* 85%, *P* < .001) at 5 years with no difference in overall survival or toxicity.^53^ Most studies for DIL boost have used a combination of T2WI plus DCE plus DWI for delineation of the DIL. However, recent studies have suggested that the actual DIL may correlate better with the volume delineated on Ga 68 prostate membrane–specific antigen PET-CT. Zamboglou et al^54^ reported the combined use of prostate membrane–specific antigen PET CT and mp-MRI for the delineation of DIL and correlated it with the tumor control probability (TCP) on the basis of histology. On average, 86% ± 10%, 74% ± 17%, and 93% ± 5% of GTV as seen on the histology specimen overlapped with PTV generated on PET, MRI, and combined PET/MRI, respectively. The plan generated using combined information from PET and MRI had significantly higher TCP values than either PET or MRI alone.

MRI as the sole imaging modality for RT treatment planning is gaining ground saving additional CT scan required for RT planning and eliminating the uncertainties from coregistration. As part of the MRI-only workflow, a pseudo-CT or synthetic CT is generated for dose computation. The methods for generating synthetic CT can be classified into voxel-based, atlas-based, and hybrid methods,^55^ with expected dose differences performed on synthetic images compared with standard CT being within 1%.^56,57^ MRI-only workflow also enables automatic delineation of prostate and OARs which can be manually adjusted. Patient setup for treatment is achieved by matching synthetic DRRs with a success rate of > 90%^58,59^ with constraints in remaining 10% because of inability to accurately identify gold fiducial markers appearing as signal void. Other issues include artifacts generated by metallic implants,^59^ large separation resulting in body contour reaching outside the FOV, and image distortion related to motion artifacts.

Interfraction and intrafraction variation of the prostate during RT has significant dosimetric and clinical implications. The use of MR-Linac can improve accuracy of delivery and combine it with real-time adaptive planning. Online matching of prostate using MRI is more accurate and thus can also be a factor in reducing PTV margins.^60^ The use of cine-MRI during beam delivery affords the option to intervene in the event of extreme anatomical changes. Using motion monitoring and gating, it has been reported that 2D shifts during treatment are required in > 20% of all delivered fractions.^61^ Table 2 shows selected studies highlighting the role of MRI in various aspects of RT for prostate cancer.

**TABLE 2:**
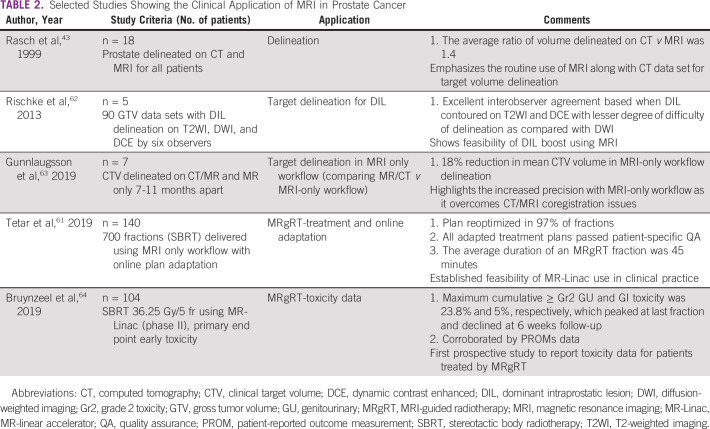
Selected Studies Showing the Clinical Application of MRI in Prostate Cancer

Multiple prospective studies exploring the utility of MR-Linac for prostate RT are currently underway. Recently, the interim analysis from the phase III randomized MIRAGE study was presented^65^ comparing SBRT for localized prostate cancer (40 Gy/5 fr) using CT versus MRI guidance. The primary end point was acute grade ≥ 2 GU toxicity within 90 days. One hundred patients were evaluated (51 CT and 49 MRI arm). MRI-guided SBRT had significantly lower acute grade ≥ 2 GU and GI toxicity (22.4% *v* 47.1%, *P* = .01 and 0 *v*s 13.7%, *P* = .01, respectively). Patient-reported outcome in the form of EPIC-26 bowel domain scores was also in favor of the MRI-guided SBRT arm. Of note, the PTV margins used for MRI guidance were smaller than those for CT guidance (2 mm and 4 mm, respectively) and could have contributed to the large difference in the effect between the two arms.

### GI Malignancies

#### Rationale and parameters.

MRI has had a far-reaching impact in the management of GI malignancies right from staging, RT treatment planning, and execution to follow-up. MRI for rectal cancer should be performed using a 1.5-T or 3-T scanner with phased array coil positioned from the sacral promontory to 10 cm below the pubic symphysis.^66^ The use of an endorectal coil is not beneficial.^67^ The standard rectal MRI protocol includes 2D FSE T2-weighted non-fat–suppressed sequences. The sagittal series from one pelvic sidewall to another locates the tumor. Axial images are taken with a large FOV covering the entire pelvis and a smaller FOV with < 3 mm slice thickness axial and coronal to the long axis of the tumor. For low-lying rectal tumors, high-resolution coronal images are used to demonstrate levator muscles, sphincter complex, and intersphincteric plane. These sequences have high accuracy for identifying invasion of adjacent organs and mesorectal fascia and for extramural vascular invasion.^67^

Traditionally, for liver lesions, multiphase CT or MRI is used for imaging. MRI has been shown to have higher sensitivity compared with CT scan for the diagnosis of hepatocellular carcinoma, especially for lesions < 1 cm.^68^ The minimum specifications for liver MRI include the use of a minimum 1.5-T scanner with phased array torso coil. The minimum sequences to be acquired are T2-weighted (with and without fat saturation), T1-weighted in- and out-of-phase images, dynamic postcontrast gadolinium T1-weighted gradient echo sequence (3D preferable), and preferably DWI. The dynamic sequences would include late arterial phase (30-35 seconds postcontrast), portal venous phase (60-70 seconds postcontrast), and delayed phase (3-5 minutes) with < 5 mm slice thickness.^69^ Patients need to hold breath similarly for each sequence. The addition of DWI increases the detection rates, especially for smaller tumors.^70^ There is also emerging use of hepatobiliary-specific contrast agents such as gadoxetate disodium, which is progressively transported into hepatocytes and excreted through the bile ducts.

#### Clinical applications.

MRI is becoming a cornerstone in the RT planning process for upper abdominal tumors. Voroney et al compared MRI and CT-derived target volumes for liver tumors (primary and metastases) and found significant differences in the median percentage surface area difference. The median values for the percentage of surface area differing by 3 mm and 5 mm in spatial position between CT-GTV and MRI-GTV were 55% and 26%, respectively, with certain tumor foci visible only on MRI.^71^ Pech et al^72^ showed that the volume of liver metastases contoured using MRI was significantly larger than that on CT, with the difference between the target volumes being 181% for T1w images, 178% for contrast-enhanced T1w, and 246% for T2w sequences.

MRI-based target volume delineation in rectal cancer has been studied in a limited number of patients, and it has been shown that the MRI-derived volume is smaller than CT with significant differences when anal canal and sigmoid are involved^73^ (Figs 2E and 2F). Issues with CT and MRI coregistration owing to bladder filling and rectal distention at the time of the two scans preclude the routine use of MRI for external beam radiotherapy planning in rectal cancers. The best use of MRI for RT planning in rectal cancers may be for dose escalation wherein the T2 intermediate bright tumor can be accurately delineated and selectively boosted to a higher dose.^74^

Managing motion of upper abdominal organs is a significant issue during RT planning and execution. Breathing-related motion artifacts during planning CT acquisition lead to incorrect target delineation, altered dosimetry, and eventually excessive PTV margins. Cine-MRI can be used to directly visualize the 3-dimensional tumor motion. Studies for liver motion using cine-MRI have demonstrated that four-dimensional CT scan underestimated motion while fluoroscopy overestimated motion relative to cine-MRI.^75^ Pancreatic motion as assessed using cine MRI ranges from 6 to 34 mm, suggesting individualized PTV margins.^76^

Table 3 summarizes selected studies highlighting the role of MRI in various aspects of RT for GI malignancies.

**TABLE 3:**
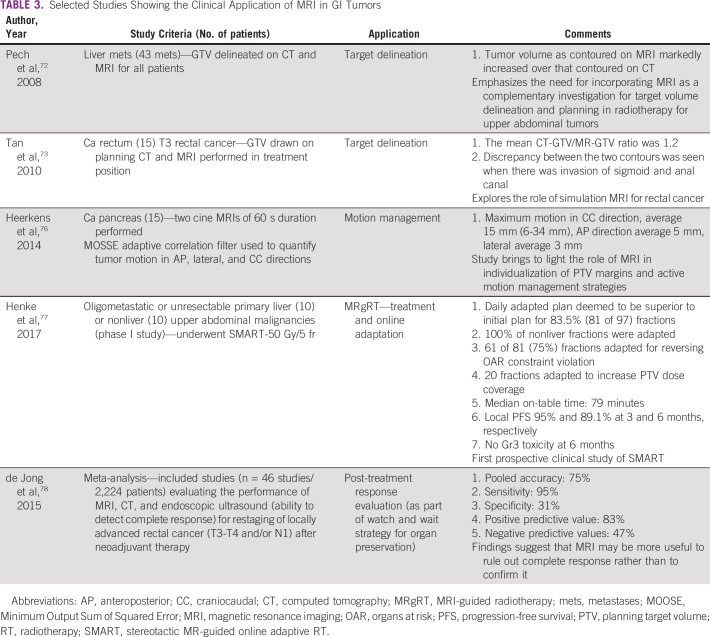
Selected Studies Showing the Clinical Application of MRI in GI Tumors

MR-guided RT is most suitable for sites where isodensity on CT does not allow discrimination of targets, especially if mobile. This makes the upper abdomen an ideal candidate for the application of this modality, considering the growing role of SBRT for primary or metastatic liver tumors and pancreatic cancers to improve therapeutic ratio. A panel of radiation oncologists and radiologists with experience in MRgRT have published an atlas for OAR contouring of upper abdomen.^79^ Peristalsis-related motion artifacts create difficulties for delineation in MR online workflow. However, drinking a glass of water shortly before the treatment fraction may help in visualizing structures, and antiperistaltic agents may reduce motion artifacts. Continuous real-time 2D cine-MRI is used to monitor target motion, thus obviating the need for implanting fiducial markers.^80^ Changes in stomach filling and bowel distention in close proximity to targets call for online adaptation, especially for peripheral liver tumors and pancreatic lesions. There are two kinds of adaptive workflows: adapt to shape and adapt to position. Adapt to shape entails an adaptation of structures as seen on the day of treatment, whereas adapt to position refers to an isocenter shift because of the inability to shift the couch on the 1.5-T MR-Linac. Henke et al demonstrated the use of MRgART in their study of SBRT (50 Gy/5 fr) for metastases or unresectable abdominal tumors, wherein all constraints were met on initial radiation planning. However, for 81 of the 97 fractions, a daily adapted plan was deemed superior. Three quarters of the plans were adapted because of violation of an OAR constraint while the rest were performed to improve target coverage. No ≥ grade 3 toxicities were observed in the 15-month follow-up period.^77^

MRI in conjunction with sigmoidoscopy for response evaluation after neoadjuvant chemoradiation in rectal cancers has heralded the wait and watch policy for patients with complete clinical response.^81,82^

Table 4 summarizes selected ongoing studies on MR-Linac for brain, prostate, and GI malignancies.

**TABLE 4:**
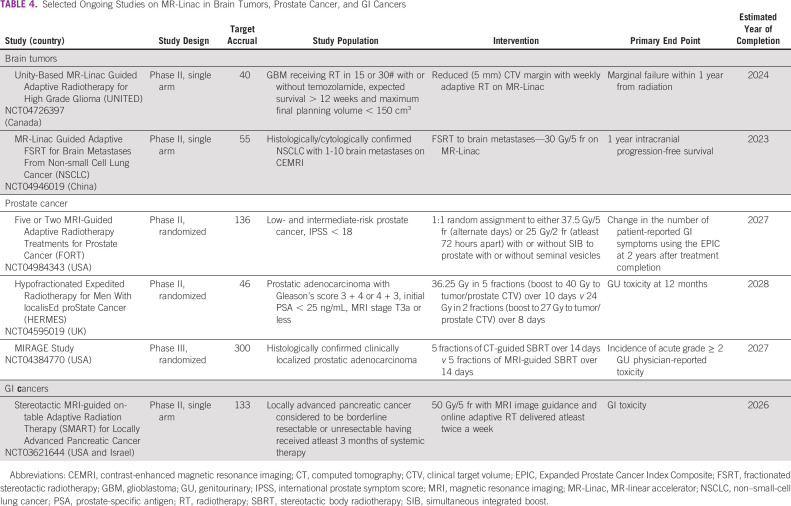
Selected Ongoing Studies on MR-Linac in Brain Tumors, Prostate Cancer, and GI Cancers

## FUTURE DIRECTIONS

**FIG 3:**
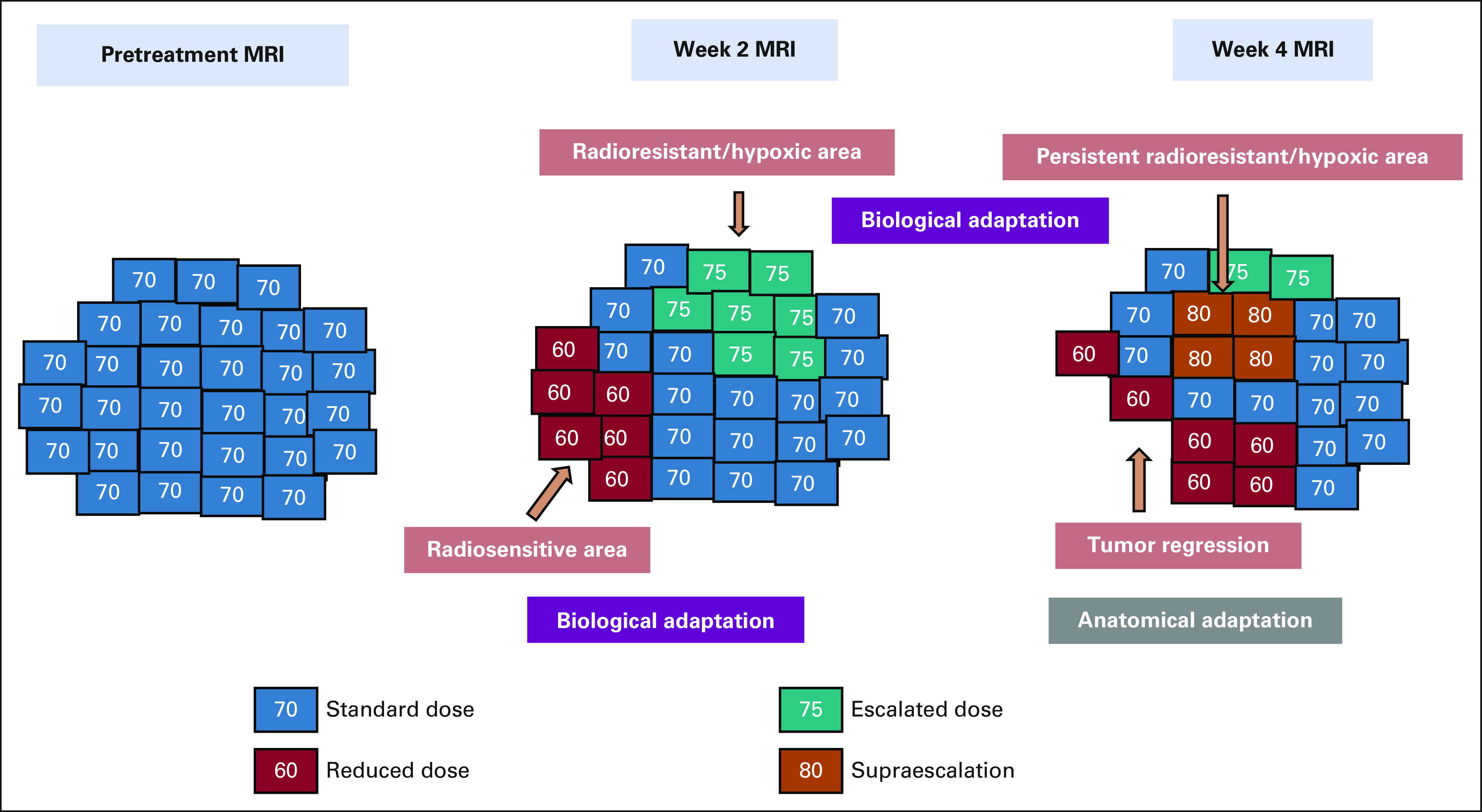
Schematic workflow for potential MRI-guided adaptive radiotherapy undertaken in a course of fractionated RT MRI is performed at week 2 and week 4 during radiation. Anatomical adaptation is undertaken for morphological changes as detected on midtreatment MRI for target volumes and organs at risk. Biological adaptation with different dose levels is performed on the basis of findings from functional MRI, with areas of refractory disease escalated to a higher dose and areas with response de-escalated to a relatively lower dose level. MRI, magnetic resonance imaging; RT, radiotherapy.

The field of medical imaging and MRI is undergoing continuous refinements with contributions from physics, computer science, and related disciplines. Ultra high-field MRI systems using 7T have been introduced in clinical practice, and 10.5T MRI has been tested in humans recently to generate better structural and functional information from enhanced signal-to-noise and contrast-to-noise ratios.^83,84^ The contribution of artificial intelligence in quantitative analysis of medical imaging is an active area of research and is more popularly known as radiomics.^85^ With MRI used in multiple steps of radiation oncology practice, radiomic analysis is expected to have a significant impact in the future to lead the way toward personalized radiation therapy.^85,86^ One of the major advances in the recent era in the therapeutic delivery of RT has been the introduction of MR-Linac in clinical practice. As described in previous sections, the clinical application of MRgRT is still in its infancy, with two MR-Linacs introduced for patient treatment in the past 5 years. Generation of TCP and normal tissue complication probability models from daily MRgRT along with rapid contour propagation and plan optimization algorithms provide the window for real-time treatment adaptation as well as dose modification (escalation/de-escalation) for target and OARs^87,88^ (Fig 3). With ongoing conceptual refinements and applications, improving the precision of treatment delivery and better provision of adaptive RT, the actual clinical merits in toxicity reduction and/or better control rates need to be solicited in the future, compared with the available linac-based RT. Inspired by the IDEAL (Idea, Development, Exploration, Assessment, and Long-term evaluation) recommendations as described for the surgical development process,^89^ the concept of R-IDEAL framework has been introduced for radiation oncology innovations.^90^ Given the higher cost of the commercially available MR-Linacs compared with standard linear accelerators, it will be necessary to critically analyze the forthcoming evidence in the context of cost-benefit analysis, which is included in stage 3 of the R-IDEAL framework. The MR-Linac platform entails higher time on couch for the patient and demands increased human resources with the involvement of therapists, physicists, and oncologists. With artificial intelligence–based algorithms, fast and robust real-time optimization procedures, modifications of contour, and planning can potentially make the adaptive workflow more efficient and less time-consuming. Further technological advances in the accelerator device of the MR-Linac with the ability to deliver higher-energy beams, thin-width microleaf collimators are desired in the future to improve radiation conformality. Finally, MRI provides opportunities for delivery of anticancer therapies such as MR-guided focused ultrasound, which can be used to induce hyperthermia, temporary opening of the blood-brain barrier, triggering drug delivery, and microbubble (ultrasound contrast) stimulation as radiosensitizers.^91-94^

In conclusion, the contemporary practice of radiation oncology involves MRI in multiple instances including diagnosis, treatment planning, treatment delivery, midtreatment adaptation, response assessment, and surveillance. MR-Linac has been introduced in clinical practice recently is promising for real-time adaptation, improving the therapeutic ratio, although future clinical studies are warranted to establish the clinical advantages. Further developments in functional imaging sequences and quantitative imaging analysis incorporating artificial intelligence strategies are expected to have significant contributions in the future to pave the way toward precision oncology.
